# High Temperature Disrupts Organelle Distribution and Functions Affecting Meiotic Maturation in Porcine Oocytes

**DOI:** 10.3389/fcell.2022.826801

**Published:** 2022-02-18

**Authors:** Song-Hee Lee, Ming-Hong Sun, Dongjie Zhou, Wen-Jie Jiang, Xiao-Han Li, Geun Heo, Xiang-Shun Cui

**Affiliations:** Department of Animal Science, Chungbuk National University, Cheongju, South Korea

**Keywords:** temperature, oocyte, maturation, organelles, distribution

## Abstract

Heat stress (HS) has been known to cause reproductive failure in animals, especially in summer. HS severely affects the developmental potential of oocytes and leads to low fertility rates. Previous studies have reported that HS compromises embryo development in bovine oocytes, and reduces ovarian development in mice, thereby impairing reproductive function in animals. However, the effect of high temperature (HT) on the organelles of porcine oocytes is unknown. In this study, we reported that exposure to HT for 24 h (41°C) significantly decreased meiotic maturation in porcine oocytes (*p* < 0.05). Further experiments on organelles found that HT induced mitochondrial dysfunction, increased abnormal mitochondrial distribution, and decreased mitochondrial membrane potential (MMP). We also found that HT induced abnormal endoplasmic reticulum (ER) distribution and higher expression of glucose regulatory protein 78 (GRP78), suggesting that HT exposure induces ER stress. Our results also indicated that exposure to HT induced abnormal distribution and dysfunction of the Golgi apparatus, which resulted from a decrease in the expression of the vesicle transporter, Ras-related protein Rab-11A (RAB11A). In addition, we found that HT exposure led to lysosomal damage by increasing the expression of lysosome-associated membrane protein 2 (*LAMP2*) and microtubule-associated protein 1A/1B-light chain 3 (LC3). In summary, our study revealed that HT exposure disrupts organelle dynamics, which further leads to the failure of meiotic maturation in porcine oocytes.

## Introduction

Global warming is a major environmental issue that leads to climate change, which causes an increase in the annual average ambient temperature. This alteration is stressful for livestock and impairs reproductive function due to an increase in body temperatures above the physiological homeothermic point (Boni, 2019). Heat stress (HS) induced by high ambient temperature has been reported to cause a reduction in milk production in cows (Ravagnolo et al., 2000), and a decrease in food intake and body weight in pigs (Renaudeau and Noblet, 2001). In addition, HS is widely known to cause reproductive failure, especially in summer; a decrease in pregnancy rate in cows has been reported in warm seasonal period compared to cool season (warm season: from May to September; average temperature: 21.34°C, maximum temperature: 31.5°C, cool season: from October to next April; average temperature: 9.43°C, maximum temperature: 20.2°C) (Garcia-Ispierto et al., 2006). Furthermore, HS affects both sexes (Takahashi, 2012), and also negatively affects oocytes and early embryos, disrupting fertilization and embryo development (Jin et al., 2007; Hansen, 2019). This causes huge economic damage to livestock and influences the welfare of animals (Boni, 2019). Various studies have investigated and reported the effects of HS on reproductive functions and gametes.

Cytoplasmic maturation of oocytes is characterized by multiple events, including reorganization of organelles, such as mitochondria, ER, Golgi apparatus, and lysosomes, and storage of mRNA, proteins, and transcription factors required for oocyte maturation (Watson, 2007). Proper spatial and temporal dynamics of organelles ensure high developmental potency of oocytes (Sirard et al., 2006). Mitochondria are organelles that produce ATP, which plays an essential role in cellular metabolism, homeostasis, and cell survival (Roth, 2018). Mitochondria on close contact with a specialized domain of the ER form the mitochondria-associated ER membranes, which play key roles in cellular processes (Guzel et al., 2017). ER is the major internal storage site for calcium ions, maintains calcium homeostasis, and is also involved in protein folding and degradation, and lipid metabolism (Mao et al., 2014). In particular, protein synthesis and transport are necessary for multiple biological events for meiotic resumption in oocytes (Guzel et al., 2017). Newly synthesized molecules from the ER are transported to the Golgi apparatus through the secretory pathway (Passemard et al., 2019). The Golgi apparatus plays a central role in intracellular trafficking processes involved in protein transport and modification (Mao et al., 2014). To regulate cellular homeostasis, cellular waste is recycled in lysosomes through the autophagy pathway, which sequesters damaged organelles and misfolded proteins for lysosomal degradation (Ballabio and Bonifacino, 2020).

Several factors, such as maternal feeding, management, stress, and various environmental factors, affect oocyte developmental competence (Belhadj Slimen et al., 2016). Oocyte quality is a key factor affecting embryo development (Eppig et al., 2002), and it is often decreased due to mitochondrial dysfunction (Sun et al., 2020). It has been reported that mitochondrial distribution is disrupted more in summer compared to other seasons (Gendelman and Roth, 2012). Exposure to high temperature (41°C) during the first 6 h increases mitochondrial reactive oxygen species (ROS) in bovine oocytes (Payton et al., 2018). Oxidative stress by ROS causes DNA damage and mitochondrial dysfunction that leads to apoptosis (Patwardhan et al., 2016). In addition, previous studies have shown that HS affects ER function (Li et al., 2017), and induces Golgi disorganization and hastens Golgi fragmentation in Panc-1 cells (Petrosyan and Cheng, 2014). Moreover, some studies have reported that heat stroke induces autophagy and impairs lysosomal function in mice (Yi et al., 2017).

Over the past few decades, there has been a consistent increase in environmental temperature due to global warming; in recent times, the incidence of heat waves, heavy rains, and drought has been on the rise (Takahashi, 2012). Therefore, we suspect that sustained thermal stress will affect reproductive functions in animals. Although previous studies have shown that HS compromises oocyte maturation and developmental competence (Roth, 2018), little is known about the mechanisms through which it disturbs mitochondrial function and distribution in oocytes and cumulus cells (Paula-Lopes et al., 2013). Nevertheless, the impact of high temperature (HT) on the organelles in porcine oocytes is unknown. Thus, we aimed to explore the effect of HT exposure on distribution and function of organelles in porcine oocyte undergoing meiosis. And oocytes were treated to HT at 41°C for 15 h (0–15) or 24 h (0–24), respectively, and were investigated in metaphase I and metaphase II stage. Our results showed that HT exposure for 24 h induced mitochondrial dysfunction and abnormal distribution, which may have contributed to ER stress and abnormal ER distribution. These dysfunctions further contributed to Golgi apparatus dysfunction and affected lysosome function in porcine oocytes. Therefore, our study demonstrated the negative effect of HS on organelle functions in mammalian oocytes.

## Materials and Methods

### Chemicals and Antibodies

Mouse anti-Tom20 antibody (Cat# sc-17764) and rabbit anti-GAPDH antibody (Cat# sc25578) were obtained from Santa Cruz Biotechnology (Dallas, TX, United States). Rabbit anti-GRP78 antibody (Cat# ab21685) was purchased from Abcam (Cambridge, United Kingdom). Rabbit anti-RAB11A antibody (Cat# 2413), rabbit anti-LC3B (D11) XP (Cat# 3868), and rabbit anti-LC3 (Cat# 2775) antibody were purchased from Cell Signaling Technology (Danvers, MA, United States). Goat anti-rabbit peroxidase-conjugated secondary antibody (Cat# GTX213110-01) was purchased from Gene Tex (Irvine, CA, United States). Alexa Fluor 488 goat anti-rabbit (Cat# A32731), Alexa Fluor 488 donkey anti-mouse (Cat# A-21202), Alexa Fluor 546 donkey anti-rabbit antibodies (Cat# A10040), MitoTracker Red CMXRos (Cat# M7512), and MitoProbe JC-1 assay kit (Cat# M34152) were purchased from Invitrogen (Carlsbad, CA, United States). ER-Tracker Green, Golgi-Tracker Red, and Lyso-Tracker Red were purchased from Beyotime Biotechnology (Shanghai, China). Unless noted otherwise, all chemicals were purchased from Sigma-Aldrich (St. Louis, MO, United States).

### Oocyte Collection and *In-Vitro* Maturation

Porcine ovaries were obtained from a local slaughterhouse, and transported to the laboratory in saline at 37°C. Cumulus oocyte complexes (COCs) were aspirated from ovarian follicles that were 3–6 mm in diameter, and oocytes surrounded by a minimum of three layers of cumulus cells were selected for use in experiments. After washing thrice with Tyrode lactate HEPES (TL-HEPES) buffer, the COCs were transferred to an *in vitro* maturation (IVM) medium containing TCM-199 (Invitrogen, Carlsbad, CA, United States), supplemented with 10% (v/v) porcine follicular fluid, 0.91 mM sodium pyruvate, 0.6 mM L-cysteine, 10 ng/ml epidermal growth factor, 10 μg/ml luteinizing hormone, and 0.5 μg/ml follicle-stimulating hormone, and were incubated at 38.5°C in a humidified 5% CO_2_ incubator. In the treatment group, COCs were cultured at 41°C for 15 (0–15) or 24 (0–24) h under the same conditions as the control group, and then temperature was returned to 38.5°C for cell maturation (Jin et al., 2007). After maturation, cumulus cells were removed by gentle pipetting in 1 mg/ml hyaluronidase. Following this, we used oocytes in IVM medium after 24 h (metaphase I, MI) or 44 h (metaphase II, MII).

### Immunofluorescence Staining

Immunofluorescence staining was performed as described previously (Niu et al., 2020; Zhou et al., 2020). After washing three times with PBS containing polyvinyl alcohol (PVA), the oocytes were fixed with 3.7% formaldehyde at room temperature (RT) for 30 min. The oocytes were then permeabilized in 0.5% Triton X-100 at RT for 30 min, and blocked with 3% BSA in PBS/PVA at RT for 1 h. Subsequently, the oocytes were incubated overnight at 4°C with mouse anti-Tom20 antibody (1:100), rabbit anti-GRP78 antibody (1:100), rabbit anti-Rab11a antibody (1:20), and rabbit anti-LC3 antibody (1:100). After three washes with PBS/PVA, the oocytes were incubated with goat anti-rabbit IgG (1:100), donkey anti-mouse IgG (1:100), or donkey anti-rabbit IgG (1:100) at 37°C for 1 h. Then, the oocytes were incubated for 10 min with 5 μg/ml Hoechst 33,342 dye. Finally, the oocytes were mounted on slides and examined under a confocal microscope (Zeiss LSM 710 META, Oberkochen, Germany). Images were obtained using the Zen software v.8.0 (Zeiss, Jena, Germany), and then analyzed using the ImageJ software (National Institutes of Health, Bethesda, MD, United States).

### Mitochondria, ER and Lysosome Distribution Detection

To explore the distribution of mitochondria, ER, and lysosomes, oocytes were incubated with MitoTracker Red CMXRos (1:500), ER-Tracker Green (1:20), or Lyso-Tracker Red (1:10,000) in IVM at 38.5 °C for 30 min. After washing three times with fresh culture medium, the signal from each oocyte was detected using a confocal microscope (Zeiss LSM 710 META).

### Golgi Apparatus Detection

First, we incubated oocytes with 1% pronase for 3 min to remove the zona pellucida, and then incubated them with Golgi-Tracker Red (1:20) in IVM medium for 45 min at 4°C. After three washes with fresh culture medium, we incubated the oocytes in IVM medium at 38.5°C for 30 min and then immediately detected the signals using a confocal microscope (Zeiss LSM 710 META).

### Active Mitochondrial Staining

Oocytes that were cultured for 24 h in IVM medium were incubated with MitoTracker Red CMXRos (1:500) at 38.5°C for 30 min. After washing three times with fresh IVM medium, oocytes were stained with Tom20 as described previously in the immunofluorescence staining section under Materials and Methods.

### Mitochondrial Membrane Potential Assay

Denuded oocytes were incubated in IVM medium containing 2.5 μM 5,5′,6,6′-tetrachloro-1,1′,3,3′-tetraethylimidacarbocyanine iodide (JC-1) at 38.5°C in a 5% CO_2_ incubator for 30 min. Membrane potentials were determined as the ratio of red fluorescence to green fluorescence, corresponding to activated mitochondria (J-aggregates) and less-activated mitochondria (J-monomers), respectively. Fluorescence was visualized using an epifluorescence microscope (Nikon, Tokyo, Japan).

### Quantitative Reverse-Transcription Polymerase Chain Reaction

mRNA was extracted from 35 COCs after 24 h of culture in IVM medium using a Dynabeads mRNA Direct Kit (61,012; Thermo Fisher Scientific, Waltham, MA, United States), and cDNA was synthesized using the First Strand Synthesis Kit (cat# 6210; LeGene, San Diego, CA, United States) in accordance with the manufacturer’s instructions. The primer sequences for amplification of cDNA were the same as those used in previous studies (Han et al., 2016; Park et al., 2018). qRT-PCR was performed using a WizPure qPCR Master (W1731-8; Wizbio Solutions, Seongnam, South Korea) according to the manufacturer’s instructions, on a QuantStudio™ six Flex Real-Time PCR System (Applied Biosystems, Waltham, MA, United States). The PCR conditions were as follows: initial denaturation at 95°C for 10°min, followed by 40 cycles of amplification at 95°C for 15°s, 60°C for 20°s, and 72°C for 15°s, and a final extension at 95°C for 15°s. Relative gene expression was calculated using the ∆∆CT method. The primers used are listed in Table 1.

**TABLE 1:** Primer sequences used in RT-qPCR.

Gene	Gene accession no.	Primer sequence (5′-3′)	Annealing Temp(°C)	Length (bp)
***Gapdh***	AK234838	F: AAG​TTC​CAC​GGC​ACA​GTC​AAG	60	112
		R: CAC​CAG​CAT​CAC​CCC​ATT​T		
***BiP*** (***GRP78***)	J03214.1	F: ACC AAT GAC CAA AAT CGC CT	60	246
		R: GTG ACT TTC CAG CCA CTC AA		
***ATF4***	NM_001123078.1	F: TGA GCC CTG ACT CCT ATC TG	60	277
		R: TCC AGC TCT TTA CAT TCG CC		
***CHOP***	NM_001144845.1	F: AAG ACC CAG GAA ACG GAA AC	60	261
		R: TCC AGG AAA GGT CAG CAG TA		
***Lamp2***	AK235422	F: GCTTTTGCAGCGTTGTGG	60	169
		R: GAC​GAG​GCA​GAG​CAT​AAG​GAG		
***Lc3***	NM_001190290	F: CCG​AAC​CTT​CGA​ACA​GAG​AG	60	206
		R: AGG​CTT​GGT​TAG​CAT​TGA​GC		
***ATG7***	AK240528	F: AGA​TTG​CCT​GGT​GGG​TGG​T	60	140
		R: GGG​TGA​TGC​TGG​AGG​AGT​TG		

### Western Blotting

One-hundred twenty oocytes were collected in sodium dodecyl sulfate sample buffer, and heated at 95°C for 5 min. Proteins were separated by sodium dodecyl sulfate-polyacrylamide gel electrophoresis, and electrically transferred to polyvinylidene fluoride membranes. The membranes were blocked with 5% skim milk in Tris-buffered saline (TBS) for 1°h, and then incubated overnight at 4°C with rabbit anti-GRP78 antibody (1:1,000), rabbit anti-Rab11a antibody (1:1,000), rabbit anti-LC3B antibody (1:1,000), and rabbit anti-GAPDH antibody (1:1,000). After washing thrice with TBS-T, the membranes were incubated with goat anti-rabbit peroxidase-conjugated secondary antibody (1:2,000) at room temperature for 1 h. Finally, the membranes were processed using SuperSignal West Femto Maximum Sensitivity Substrate (Thermo Fisher Scientific). The band intensity was determined using ImageJ software.

### Statistical Analysis

Each experiment was repeated at least three times. Data were evaluated using GraphPad Prism six software (GraphPad, San Diego, CA, United States), and statistical comparisons were made using independent sample *t*-test. Differences were considered significant at *p* < 0.05.

## Results

### Effect of HT on Meiotic Maturation in Porcine Oocytes

**FIGURE 1:**
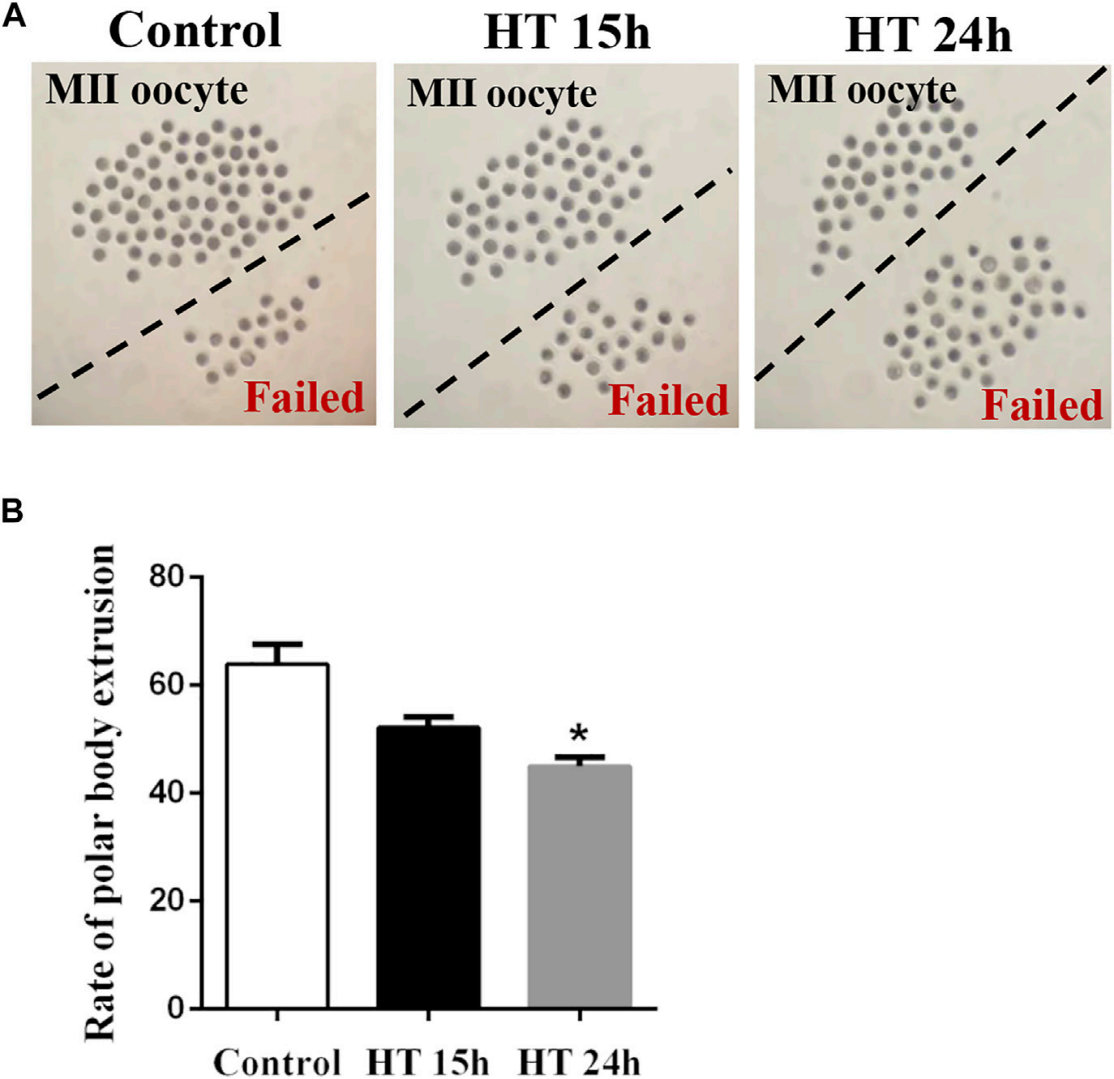
Effect of high temperature (HT) on meiotic maturation in porcine oocytes. **(A)** Representative images of the oocytes polar body extrusion in the control group and groups exposed to HT for 15 and 24 h. **(B)** The rate of polar body extrusion after HT exposure in porcine oocytes. *, *p* < 0.05.

To evaluate the harmful effects of HT on porcine oocytes, we first investigated the maturation of oocytes incubated at 41°C for 15 h or 24 h. There was no significant difference between the 15 h treated group (52.19 ± 1.88%, *n* = 270) and the control (64.59 ± 3.22%, *n* = 287, *p* > 0.05). However, after exposure to HT for 24°h, the proportion of MII oocytes decreased significantly (41.96 ± 2.93%, *n* = 273, *p* < 0.05) compared to the control group (Figure 1). These results indicated that HT exposure decreased the quality of porcine oocytes, resulting in a decrease in meiotic maturation, in a time-dependent manner. Therefore, in subsequent studies, the 24 h time point was used.

### Effect of HT on Mitochondrial Distribution and Functions in Porcine Oocytes

**FIGURE 2:**
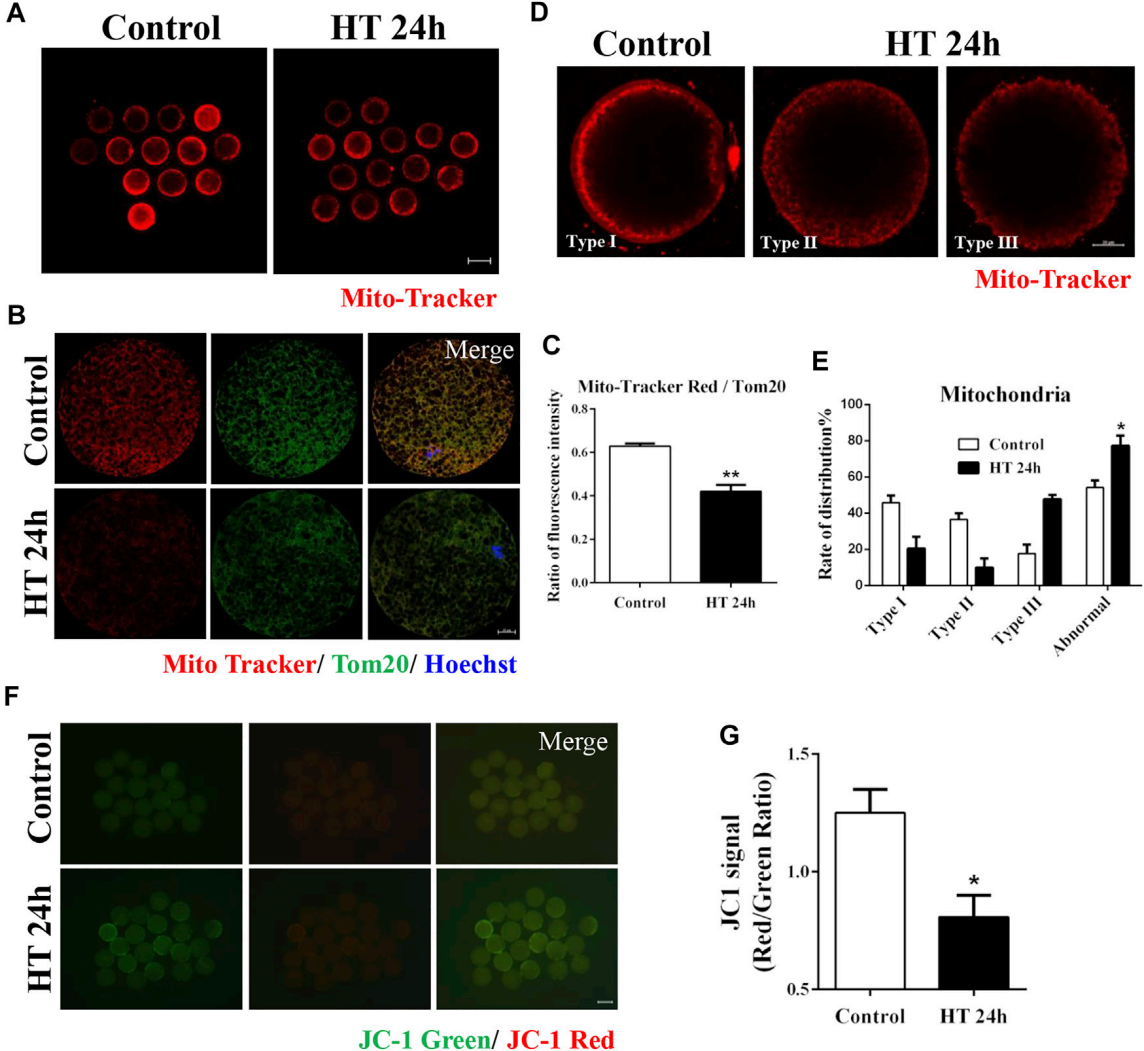
Effect of HT on mitochondrial distribution and function in porcine oocytes. **(A)** Representative images of functional mitochondria after HT exposure in porcine oocytes. Red, Mito-Tracker. Bar = 100 µm. **(B)** Representative images of Mito-Tracker and Tom20 after HT exposure in porcine oocytes, corresponding to functional mitochondria and total mitochondria, respectively. **(C)** The ratio of Mito-Tracker/Tom20 after HT exposure in porcine oocytes. Red, Mito-Tracker; Green, Tom20. Bar = 20 µm **, *p* < 0.01. **(D)** Representative images of the mitochondria distribution after HT exposure in porcine oocytes. Type Ⅰ, cluster pattern in the submembranous area; Type Ⅱ, granule pattern in pericortical area; Type Ⅲ, crystal pattern in peripheral area. In all three patterns, no mitochondria were detected in the center of the oocyte. Abnormal distribution: Type Ⅱ, Type Ⅲ. **(E)** The rate of abnormal mitochondria distribution after HT exposure. Red, Mito-Tracker. Bar = 20 µm *, *p* < 0.05. **(F)** Representative images of the JC-1 green and red channel after HT exposure in porcine oocytes. **(G)** The JC-1 signal (red/green ratio) after HT exposure. Bar = 100 µm *, *p* < 0.05.

To investigate the effects of HT on the organelles in porcine oocytes, we first examined the mitochondria, the organelles that produce ATP, and investigated the effect of HT exposure on mitochondrial activity and distribution. As shown in Figure 2A, the fluorescence intensity of Mito-Tracker in the HT-treated group was lower than that in the control group. We also calculated the ratio of Mito-Tracker and Tom20, representing the functional mitochondria and total mitochondria, respectively, and it was significantly decreased in the HT-treated group (control group, 0.63 ± 0.01, *n* = 37; HT group, 0.42 ± 0.03, *n* = 37, *p* < 0.01, Figures 2B,C), suggesting that HT exposure decreased the number of functional mitochondria. To confirm the distribution of mitochondria in porcine oocytes, we classified the distribution into three types in porcine oocytes post-IVM. As shown in Figure 2D, Type I: the cluster pattern showed mitochondrial aggregates, and was composed of large clusters in the submembranous area of the oocyte; Type Ⅱ: the granule pattern showed a dispersed granule distribution in the pericortical area of the oocyte; Type III: the crystal pattern showed mitochondria distributed in the peripheral area of the oocyte, a crystalline dot distribution (Sha et al., 2010). We considered Type II and III patterns as abnormal distribution. The oocytes in the control group showed clustered distribution (Type Ⅰ, 45.83 ± 3.95%), granule distribution (Type Ⅱ, 36.51 ± 3.42%), and crystal distribution (Type Ⅲ, 17.65 ± 4.86%). However, the HT-treated group showed significantly higher abnormal mitochondrial distribution, i.e., crystal distribution (Type Ⅲ, 47.67 ± 2.49%) and granule distribution (Type Ⅱ, 9.91 ± 4.96%), and lower normal distribution (Type Ⅰ, 20.47 ± 6.46%) compared to the control group (Normal: control group, 45.83 ± 3.95%, *n* = 41; HT group, 20.47 ± 6.46%, *n* = 41; Abnormal: control group, 54.16 ± 3.95%, *n* = 41; HT group, 77.26 ± 5.71%, *n* = 41, *p* < 0.05, Figure 2E). To examine the effect of HT exposure on mitochondrial function, we analyzed mitochondrial membrane potential (∆ψ, MMP) using JC-1 staining. In comparison to the control group, the HT-treated group showed a significantly lower ratio of J-aggregate (red) to J-monomer (green), representing a decrease in MMP in oocytes (control group, 1.25 ± 0.10, *n* = 58; HT group, 0.81 ± 0.10, *n* = 58, *p* < 0.05, Figures 2F,G). Collectively, these results indicate that HT exposure induces mitochondrial dysfunction and abnormal mitochondrial distribution in porcine oocytes.

### Effect of HT on Endoplasmic Reticulum Distribution and Function in Porcine Oocytes

**FIGURE 3:**
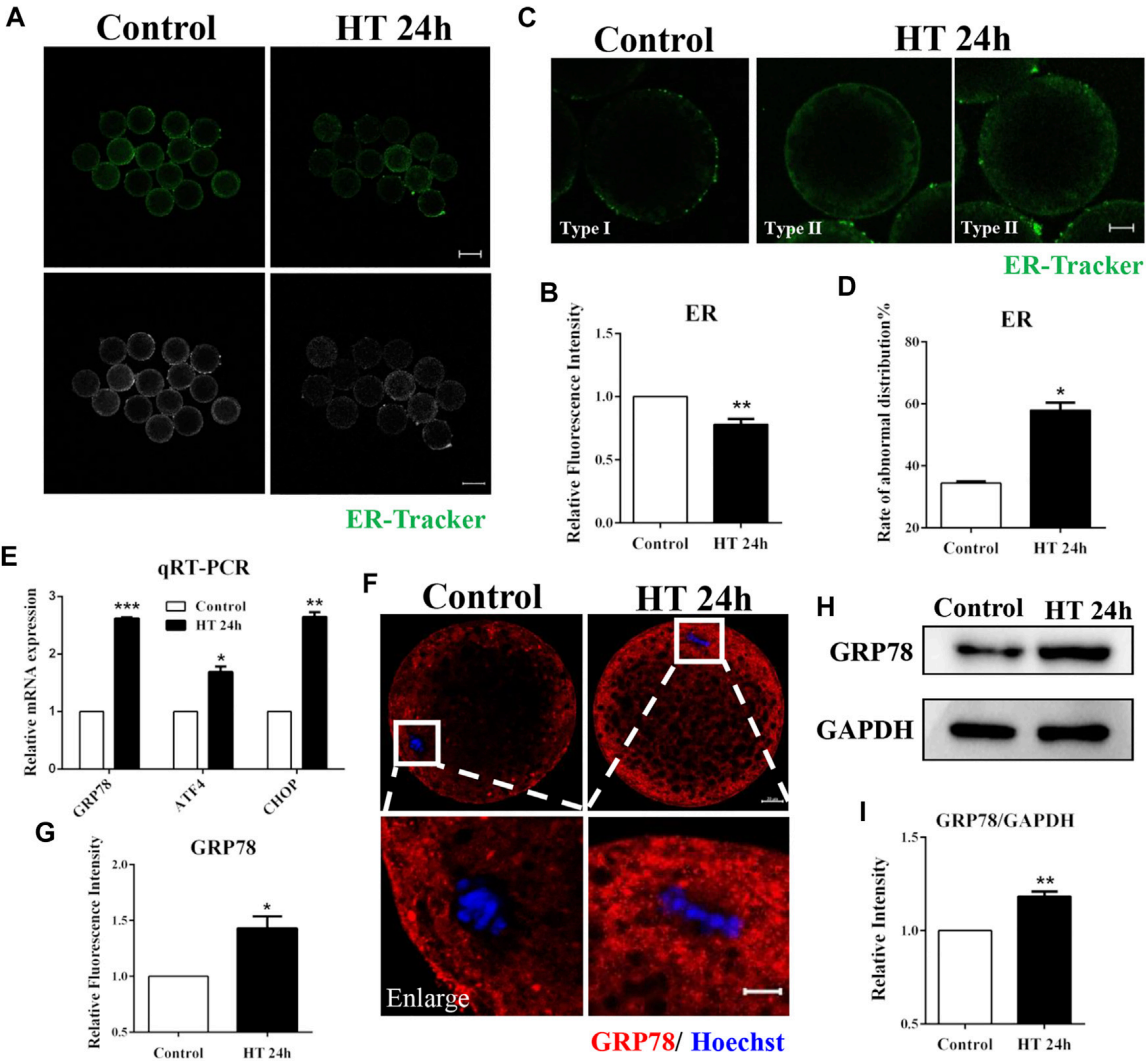
Effect of HT on ER distribution and function in porcine oocytes. **(A)** Representative images of the ER after HT exposure in porcine oocytes. **(B)** Relative fluorescence intensity of ER-Tracker after HT exposure. Green, ER-Tracker. Bar = 100 µm **, *p* < 0.01. **(C)** Representative images of the ER distribution after HT exposure in porcine oocytes. Type Ⅰ, accumulated pattern in proximity to oolemma; Type Ⅱ, diffused pattern in peripheral area, neither reached in cortex nor accumulated. Abnormal distribution: Type Ⅱ. **(D)** The rate of abnormal ER distribution after HT exposure. Green, ER-Tracker. Bar = 20 µm *, *p* < 0.05. **(E)** The relative mRNA expression of GRP78, ATF4, and CHOP in the control and HT-treated group. *, *p* < 0.05; **, *p* < 0.01; ***, *p* < 0.001. **(F)** Representative images of GRP78 intensity after HT exposure in porcine oocytes. Bar = 100 μm; enlarged bar = 20 µm. **(G)** Relative fluorescence intensity of GRP78 after HT exposure. Red, GRP78; Blue, DNA. *, *p* < 0.05. **(H)** Western blot result of the protein expression of GRP78 after HT exposure. **(I)** The band intensity analysis for GRP78 after HT exposure. **, *p* < 0.01.

The ER membrane interacts spatially and functionally with mitochondria (Guzel et al., 2017). Therefore, we investigated the effect of HT exposure on ER function and distribution using ER-Tracker. The fluorescence intensity of ER-Tracker was significantly lower in the HT-treated group in comparison to the control group (fluorescence intensity of control group was set as one fold, *n* = 48; HT group, 0.78 ± 0.04, *n* = 46, *p* < 0.01, Figures 3A,B). We also assessed ER distribution using ER-Tracker. The ER is mainly localized in the cortex after oocyte maturation, similar to the polarized distribution of cortical granules (CGs), and forms ER clusters, which are 1–2 µm in diameter (Kline, 2000). Therefore, we classified ER distribution into two types. In Type I, ER was distributed in proximity to the oolemma and formed clusters, while in Type II, ER was diffusely distributed in the peripheral area and neither reached the cortex nor was clustered. We considered Type II as abnormal distribution (Figure 3C). In the control group, most of the ER was distributed in the peripheral area of the oocyte and formed clusters (Type Ⅰ, 65.56 ± 2.40%). However, in HT-treated group, the rate of abnormal ER distribution was significantly increased (Type II: control group, 34.44 ± 0.52%, *n* = 30; HT group, 57.95 ± 2.40%, *n* = 30, *p* < 0.05, Figure 3D). To further verify the effects of HT on the ER, we investigated induction of ER stress using ER stress markers, such as glucose regulatory protein 78 (*GRP78*), activating transcription factor 4 (*ATF4*), and C/EBP homologous protein (*CHOP*). In the HT-treated group, the mRNA expression of *GRP78*, *ATF4*, and *CHOP* increased significantly after HT exposure (control group, 1, *n* = 35; *GRP78*, 2.62 ± 0.02, *n* = 35, *p* < 0.001; *ATF4*, 1.69 ± 0.09, *n* = 35, *p* < 0.05; *CHOP*, 2.64 ± 0.08, *n* = 35, *p* < 0.01, Figure 3E). We then examined the protein expression of GRP78; the fluorescence intensity of GRP78 was significantly higher in the HT-treated group (control group, 1, *n* = 26; HT group, 1.43 ± 0.11, *n* = 26, *p* < 0.05, Figure 3F,G). The relative band intensity of GRP78 was also significantly increased in HT-treated group compared to the control group (control group, 1, *n* = 120; HT group, 1.18 ± 0.03, *n* = 120, *p* < 0.01, Figure 3H,I). Thus, these results suggest that HT exposure induces abnormal ER distribution and increases ER stress in porcine oocytes.

### Effect of HT on Golgi Apparatus Distribution and Function in Porcine Oocytes

**FIGURE 4:**
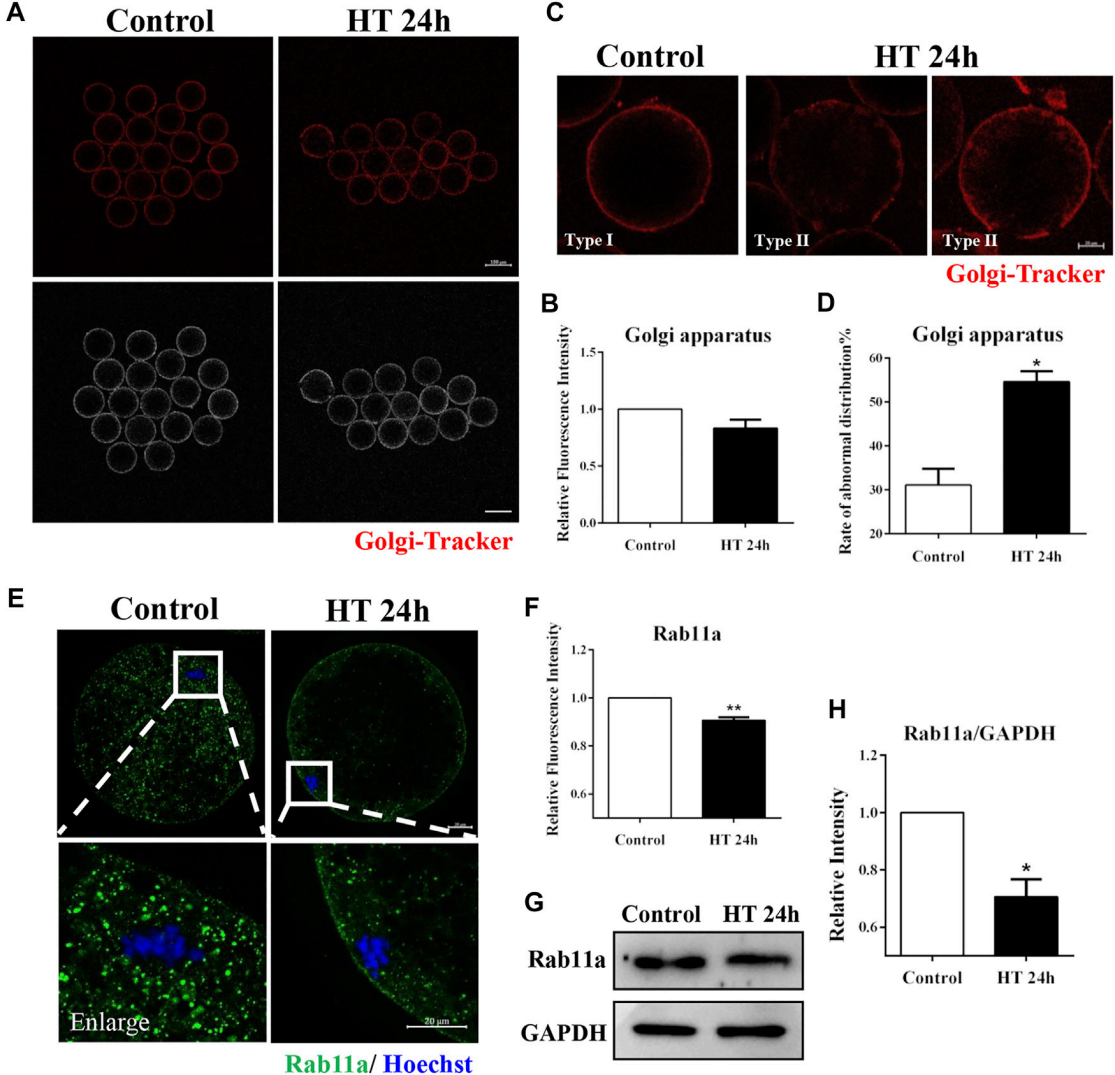
Effect of HT on Golgi apparatus distribution and function in porcine oocytes. **(A)** Representative images of the Golgi apparatus after HT exposure in porcine oocytes. **(B)** Relative fluorescence intensity of Golgi-Tracker after HT exposure. Red, Golgi-Tracker. Bar = 100 µm. **(C)** Representative images of the Golgi apparatus distribution after HT exposure in porcine oocytes. Type Ⅰ, accumulated pattern in proximity to the oolemma; Type Ⅱ, diffused pattern in the cortical area, and does not completely reach the cortex. Abnormal distribution: Type Ⅱ. **(D)** The rate of abnormal Golgi apparatus distribution after HT exposure. Red, Golgi-Tracker. Bar = 20 µm *, *p* < 0.05. **(E)** Representative images of the RAB11a intensity after HT exposure in porcine oocytes. Bar = 100 μm; enlarged bar = 20 µm. **(F)** Relative fluorescence intensity of RAB11a after HT exposure. Green, RAB11a; Blue, DNA. **, *p* < 0.01. **(G)** Western blot result of RAB11a protein expression after HT exposure in porcine oocytes. **(H)** The band intensity analysis for RAB11a after HT exposure. *, *p* < 0.05.

The Golgi apparatus mainly processes and transports proteins synthesized in the ER, and this transporter function is necessary for intercellular progress. Therefore, given the observed effect of HT exposure on ER, we investigated the effect of HT exposure on the distribution and function of the Golgi apparatus. There was no significant difference in fluorescence intensity of Golgi-Tracker between the groups (control group, 1, *n* = 45; HT group, 0.83 ± 0.08, *n* = 40, *p* > 0.05, Figure 4A,B). We also assessed the distribution of the Golgi apparatus using Golgi-Tracker in porcine oocytes post-IVM. The distribution of the Golgi apparatus is connected with the distribution of the ER and CGs, and this organelle is generally localized in the cortex of MII oocytes (De los Reyes et al., 2012). Therefore, we classified the distribution of the Golgi apparatus into two types. In Type I, fragmented Golgi apparatus was distributed in proximity to the oolemma, while in Type II, fragmented Golgi apparatus was diffused from and did not completely reach the cortex. We considered Type II as the abnormal distribution (Figure 4C). Abnormal distribution of Golgi apparatus was significantly increased in the HT-treated group compared to the control group (control group, 29.93 ± 2.96%, *n* = 47; HT group, 52.74 ± 3.68%, *n* = 43, *p* < 0.05, Figure 4D). To further identify the effects of HT on trafficking function of the Golgi apparatus, we analyzed the expression of the vesicle transporter Ras-related protein Rab-11A (RAB11A), a major regulator of membrane delivery during cytokinesis (Welz et al., 2014). The fluorescence intensity of RAB11A in the HT-treated group was significantly lower than that in the control group (control group, 1, *n* = 32; HT group, 0.91 ± 0.01, *n* = 26, *p* < 0.01, Figures 4E,F). Furthermore, the protein expression of RAB11A was significantly decreased after HT exposure (control group, 1, *n* = 120; RAB11A, 0.71 ± 0.06, *n* = 120, *p* < 0.05, Figures 4G,H). These results indicate that HT exposure led to aberrant distribution and dysfunction of the Golgi apparatus in porcine oocytes.

### Effect of HT on Lysosome Functions in Porcine Oocytes

**FIGURE 5:**
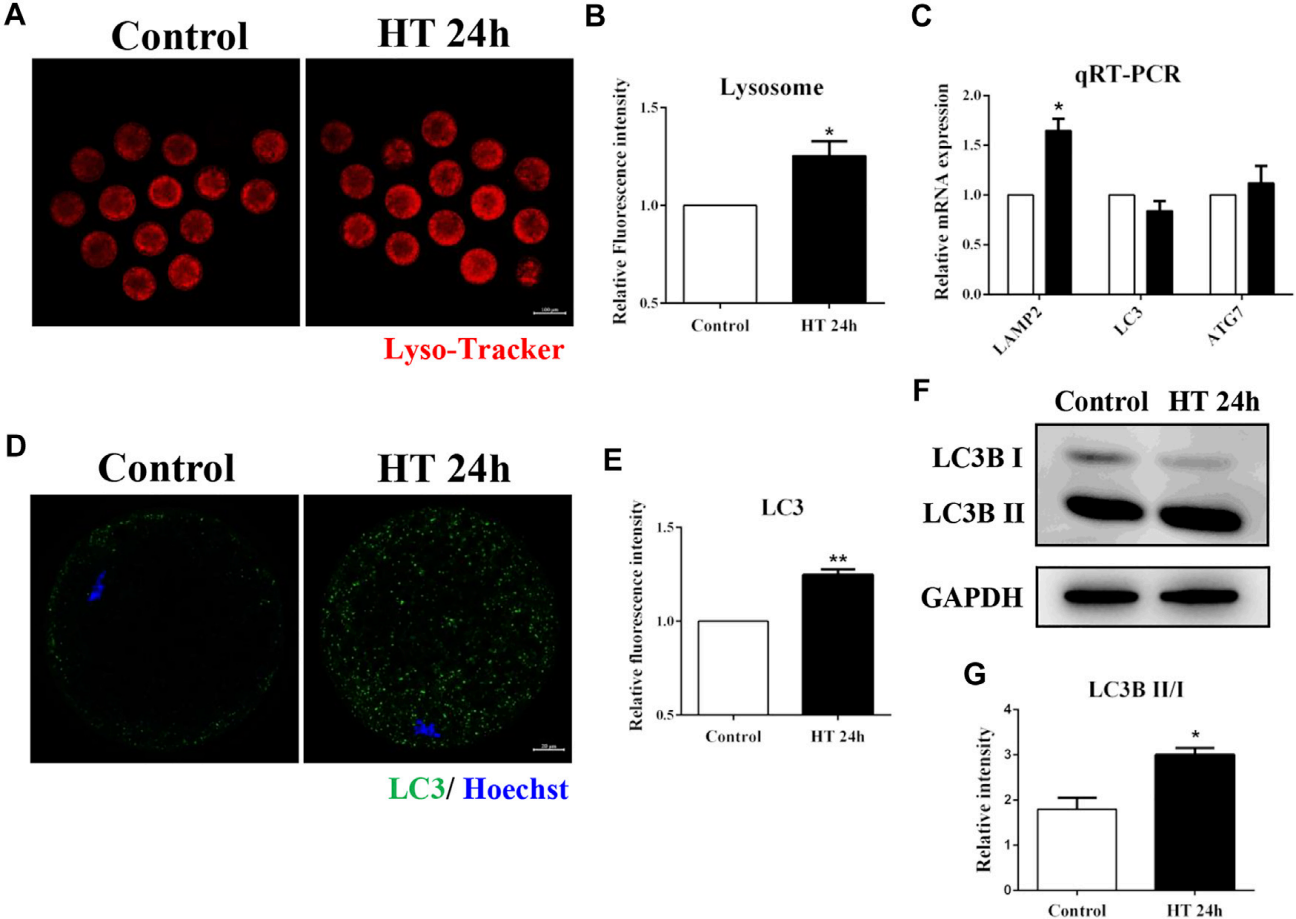
Effect of HT on lysosome function in porcine oocytes. **(A)** Representative images of lysosomes after HT exposure in porcine oocytes. **(B)** Relative fluorescence intensity of Lyso-Tracker after HT exposure. Red, Lyso-Tracker. Bar = 100 µm *, *p* < 0.05. **(C)** The relative mRNA expression of LAMP2, LC3, and ATG7 in the control and HT-treated group. *, *p* < 0.05. **(D)** Representative images of LC3 intensity after HT exposure in porcine oocytes. **(E)** Relative fluorescence intensity of LC3 after HT exposure. Green, LC3; Blue, DNA. Bar = 20 µm **, *p* < 0.01. **(F)** Western blot result of LC3B-Ⅱ/Ⅰ protein expression after HT exposure in porcine oocytes. **(G)** The band intensity analysis for LC3B-Ⅱ/Ⅰ ratio after HT exposure. *, *p* < 0.05.

Lysosomes are derived from vesicles of the Golgi apparatus, and participate in intracellular trafficking (Bajaj et al., 2019), and are also involved in the autophagy pathway which recycles damaged organelles (Ballabio and Bonifacino, 2020). Given the effect of HT exposure on the Golgi apparatus, mitochondria, and ER, we investigated the effect of HT exposure on lysosomal function using Lyso-Tracker. The fluorescence intensity of Lyso-Tracker was significantly higher in the HT-treated group than in the control group (control group, 1, *n* = 41; HT group, 1.25 ± 0.07, *n* = 40, *p* < 0.05, Figures 5A,B). Next, we investigated the mRNA expression of lysosome-associated membrane protein 2 (*LAMP2*), a lysosomal marker protein, and the expression of autophagy related markers, microtubule-associated protein 1A/1B-light chain 3 (*LC3*), and autophagy-related 7 (*ATG7*). As shown in Figure 5C, the mRNA expression of *LAMP2* in the HT-treated group was significantly higher than in the control group (*LAMP2*: control group, 1, *n* = 35; HT group, 1.65 ± 0.12, *n* = 35, *p* < 0.05). However, there was no difference in the expression of *LC3* and *ATG7* between the groups (*LC3*: control group, 1, *n* = 35; HT group, 0.84 ± 0.10, *n* = 35; *ATG7*: control group, 1, *n* = 35; HT group, 1.12 ± 0.17, *n* = 35). Next, we examined the expression of autophagy-related protein, LC3, in porcine oocytes. The fluorescence intensity of LC3 was significantly increased after HT exposure (control group, 1, *n* = 30; HT group, 1.25 ± 0.03, *n* = 27, *p* < 0.01, Figure 5D,E). We also confirmed that the relative LC3B-Ⅱ/Ⅰ ratio was significantly increased in the HT-treated group, suggesting an increase in protein expression (control group, 1.79 ± 0.26, *n* = 120; HT group, 3.01 ± 0.15, *n* = 120, *p* < 0.05, Figure 5F,G). These results demonstrated that HT exposure affected the function of lysosomes by inducing lysosomal damage in porcine oocytes.

## Discussion

**FIGURE 6:**
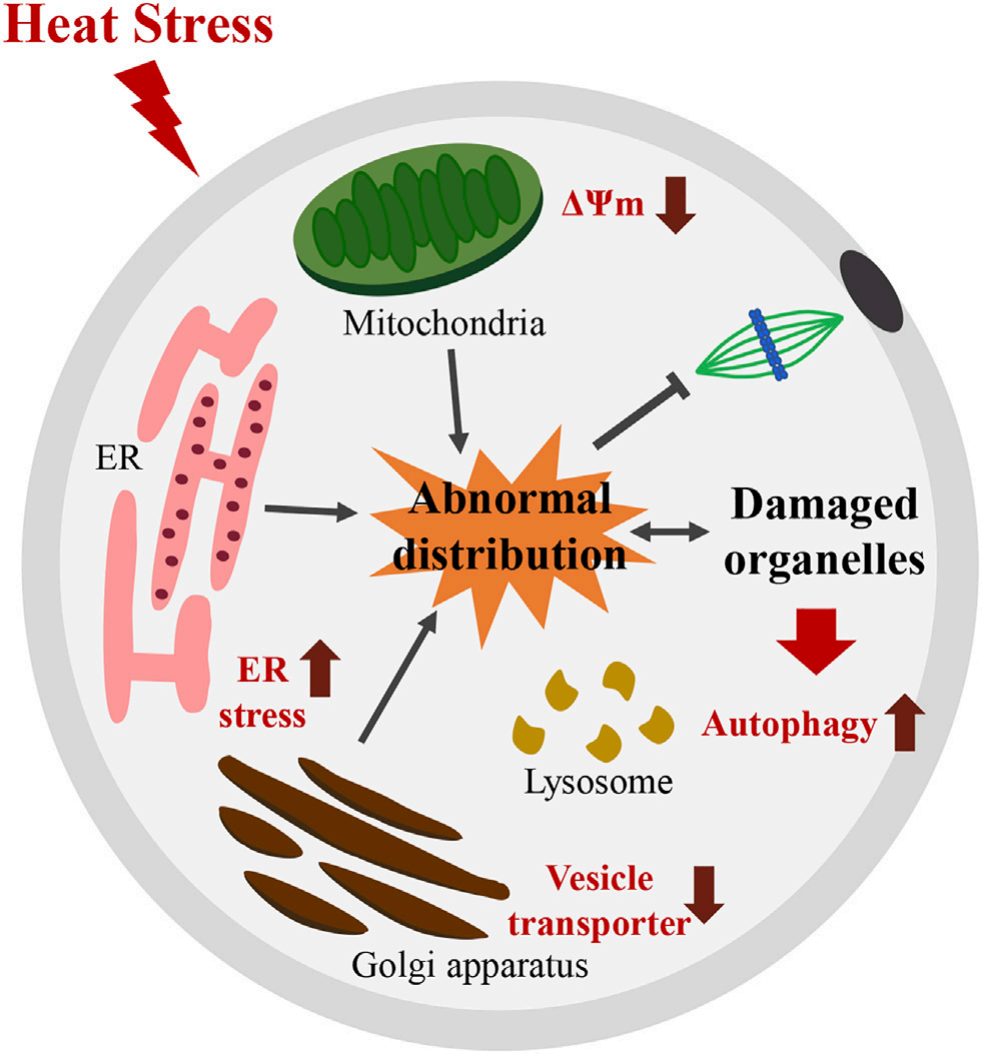
Schematic diagram illustrating how HT disturbs meiotic maturation by inducing abnormal distribution and dysfunction of organelles in porcine oocytes. HT exposure induces abnormal distribution and dysfunction of mitochondria, with a decrease in the number of functional mitochondria and in MMP. HT exposure also causes abnormal ER distribution and induced ER stress. Moreover, HT exposure induces abnormal distribution and dysfunction of Golgi apparatus, reflected by a decrease in vesicle transporters. These results indicate an increase in damaged organelles. Moreover, an increase in lysosomal damage and autophagy after HT exposure was noted, a consequence of recycling of damaged organelles. Thus, organelles defects induced by HT exposure disturb meiotic maturation in porcine oocytes. ∆ψm; MMP: mitochondrial membrane potential.

The effect of HT on reproductive functions in animals has been well described in previous studies (Garcia-Ispierto et al., 2006; Boni, 2019; Hansen, 2019). In this study, we found that HT exposure adversely affected organelle dynamics in porcine oocytes. Our results revealed that exposure to HT at 41°C for 24 h (0–24) reduced the rate of meiotic maturation in porcine oocytes. Further investigation on the effect of HT exposure on organelles revealed that HT exposure induced abnormal distribution and dysfunction of mitochondria, causing a decrease in mitochondrial activity. HT exposure also led to abnormal ER distribution, and increased ER stress. Moreover, HT exposure induced abnormal Golgi apparatus distribution and dysfunction, and a decrease in vesicle transporters. Finally, HT exposure also induced lysosomal damage and autophagy, which indicate an increase in damaged organelles (Figure 6).

Our results showed that HT exposure significantly decreased the rate of meiotic maturation in porcine oocytes, thereby reducing the first polar body extrusion. The first polar body morphology reflects oocyte competence (De Santis et al., 2005), which in turn reflects the ability of an oocyte to undergo maturation and sustain embryonic development (Eppig et al., 2002). Our results revealed that HT exposure could decrease oocyte competence. Several studies have reported that HT significantly decreased cleavage rate and blastocyst formation rate in cows (Rivera and Hansen, 2001), and decreased the total cell numbers in blastocysts in pigs (Yin et al., 2019).

To verify the effect of HT exposure on oocyte quality, we first examined the effect of HT exposure on mitochondria in porcine oocytes. Our results showed that HT exposure compromised mitochondrial distribution, decreased MMP, and eventually triggered mitochondrial dysfunction in porcine oocytes. Mitochondrial dysfunction has been reported to suppress oocyte quality (Roth, 2018). Previous studies have reported that HS elevates the amount of ROS and induces death of porcine oocytes, and decreases ATP synthesis in cumulus cells (Yin et al., 2019). Mitochondrial localization is associated with mitochondrial function and affects the development of oocytes (Dumollard et al., 2007). In this study, we classified mitochondrial distribution in post-IVM porcine oocytes into three types (Sha et al., 2010). Some studies have reported that peripheral distribution is frequently observed before IVM; however, following IVM, the peripheral pattern is rarely observed (Pawlak et al., 2016). Moreover, under normal conditions, mitochondria form large clusters around lipid droplets in the cortical area (Sha et al., 2010). Mitochondrial repositioning in the cell occurs along microtubules and actin filaments (Zampolla et al., 2011). In addition, some studies have suggested that HT exposure decreases microfilaments and microtubules in bovine oocytes (Roth and Hansen, 2005). Our results showed that HT exposure induced an increase in peripheral distribution (Type III) of mitochondria and decreased MMP, suggesting a decrease in mitochondrial activity.

The ER membrane interacts directly or indirectly with the mitochondria and Golgi bodies (Guzel et al., 2017). Given the effect of HT exposure on mitochondria, we investigated the effect of HT exposure on ER distribution and function. ER stress is defined as the excess accumulation of unfolded/misfolded proteins in the ER lumen, which activates the unfolded protein response (UPR) signaling pathways as a coping mechanism (Guzel et al., 2017). The UPR signaling pathway includes proteins, such as GRP78, ATF4, and CHOP, which are regarded as ER stress markers (Rana, 2020). Our results showed that HT exposure increased the mRNA expression of *GRP78*, *CHOP*, and *ATF4*, and increased the protein expression of GRP78, indicating the occurrence of ER stress. Similarly, studies have reported that HS increased ER stress in mouse granulosa cells (Xiong et al., 2020), and in bovine granulosa cells (Alemu et al., 2018). Following oocyte maturation, ER is redistributed and localized in the cortex, and forms clusters, which are usually 1–2 mm in diameter (Kline, 2000). Localization of ER clusters is analogous to the distribution of polarized CGs (Kline, 2000), and is essential for preparing oocytes for the generation of rapid calcium waves, which induce oocyte activation during fertilization (De los Reyes et al., 2012). Previous studies have suggested that citrinin exposure increases abnormal ER distribution in mice (Sun et al., 2020). Another study reported that HS increased the incomplete migration of CGs in ovine oocytes (Gharibzadeh et al., 2015). In our study, we showed that HT exposure not only induced abnormal ER distribution, but also increased ER stress in porcine oocytes.

The Golgi apparatus is a crucial organelle involved in the secretory process, which transports newly synthesized proteins and lipids from the ER, and is therefore often affected by ER stress (Passemard et al., 2019). Therefore, based on the results of ER distribution and function, we investigated the effect of HT exposure on distribution and function of the Golgi apparatus. Normally, the Golgi apparatus is fragmented and dispersed in the cortical area in canine MII oocytes (De los Reyes et al., 2012). The number of Golgi apparatus membranous vesicles in the cortex increases following oocyte maturation in mouse oocytes (Ducibella et al., 1988). The proper distribution of the Golgi apparatus is closely related to the ER and is associated with ER repositioning and CG exocytosis during oocyte activation (De los Reyes et al., 2012). In addition, Golgi proteins may associate with CGs, membrane-bound organelles generated by the Golgi complex, and rough ER (Selman and Anderson, 1975). In the present study, we evaluated the distribution of the Golgi apparatus in porcine oocytes post-IVM. HT exposure disrupted Golgi apparatus distribution; there was an increase in diffused distribution of Golgi apparatus from the cortex in the HT-treat group compared to the control group. Previous results indicated that HT exposure interrupted ER distribution, and HS has been reported to increase incomplete migration of CGs in ovine oocytes (Gharibzadeh et al., 2015). RAB proteins function as vital regulators of intracellular vesicular transport, and act as transport vesicles on the actin and microtubule cytoskeleton (Zou et al., 2021). Studies also indicated that citrinin exposure caused defects in RAB11A (Sun et al., 2020). Our results showed that HT exposure induced Golgi apparatus dysfunction, decreasing the expression of vesicle transporters, such as RAB11A.

Lysosomes are membrane organelles that degrade and recycle encapsulated macromolecules in the endocytosis and autophagy pathways (Ballabio and Bonifacino, 2020). Lysosomes are derived from vesicles of the Golgi apparatus, and participate in intracellular trafficking (Bajaj et al., 2019). Also, they are closely connected to the functions of the ER and mitochondria (Miao et al., 2019). Given the Golgi apparatus dysfunction observed, we examined the effect of HT exposure on lysosomal function in porcine oocytes. Our results showed that HT induced lysosomal damage, as indicated by higher expression of *LAMP2*, a lysosomal marker. In addition, we also observed an increase in LC3 protein expression after HT exposure, owing to the involvement of lysosomes in the autophagy pathway. Previous studies have suggested that HS induces autophagy in bovine oocytes (Latorraca et al., 2020), and mitochondrial defects induced by HT activated autophagy (Tamura et al., 2015). Our results indicate that HT exposure causes lysosomal dysfunction in porcine oocytes, observed as an increase in lysosomal damage.

In conclusion, our study demonstrated that HT exposure disturbs the distribution and functions of organelles in porcine oocytes, including mitochondria, ER, Golgi apparatus, and lysosomes. These results indicate an increase in damaged organelles following HT exposure. These abnormal organelles are induced autophagy, which further lead to the defects of meiotic maturation in porcine oocytes. However, the profound explanation for the alteration of the distribution after HT exposure still needs further mechanism study. Given present study on organelles, we expect that the results will be contributes to HS research of cells.

## Data Availability

The original contributions presented in the study are included in the article, further inquiries can be directed to the corresponding author.
